# Exogenous proline stimulates type I collagen and HIF-1α expression and the process is attenuated by glutamine in human skin fibroblasts

**DOI:** 10.1007/s11010-017-3069-y

**Published:** 2017-05-19

**Authors:** Lukasz Szoka, Ewa Karna, Kornelia Hlebowicz-Sarat, Jacek Karaszewski, Jerzy A. Palka

**Affiliations:** 1 Department of Medicinal Chemistry, Medical University of Bialystok, Mickiewicza 2 D, 15-222 Bialystok, Poland; 2 Laboratory of Cosmetology, Medical University of Bialystok, Akademicka 3, 15-267 Bialystok, Poland; 3 Department of Urology, Medical University of Bialystok, M. Skłodowskiej-Curie 24A, 15-276 Bialystok, Poland

**Keywords:** Proline, Glutamine, POX, Collagen biosynthesis, Prolidase

## Abstract

Abundance of proline (Pro) in collagen molecule led us to investigate whether Pro supply affects collagen biosynthesis in human skin fibroblasts. Treatment of the cells with milimolar concentrations (5 and 10 mM) of Pro for 24 and 48 h contributed to increase in α_1_ subunit of collagen type I (COL1A1) expression in both cells and culture medium. However, the effect was more pronounced in glutamine-free medium. In such condition, Pro induced collagen expression by about twofold in the cells, while in the medium only by about 30% during 24 h incubation, compared to control. In the presence of glutamine (Gln), exogenous Pro stimulated intracellular collagen expression only by about 30% during 24 h of fibroblasts incubation, and it was not accompanied by adequate increase of collagen secretion into medium. Gln alone stimulated the processes by about 2–3 fold during the course of the experiment. Pro-dependent increase in collagen expression in Gln-free medium was accompanied by increase in prolidase activity and expression of pAkt. In both Gln-free medium and Gln-supplemented medium, Pro induced expression of p53 and HIF-1α. The data suggest that availability of Gln, as a substrate for Pro biosynthesis, determine the utilization of exogenous Pro for the collagen biosynthesis.

## Introduction

Collagen is the most abundant protein in the body comprising 30% mass of all proteins. Type I collagen is the main one among about thirty discovered collagenous proteins. It contains about 20% of proline (Pro) and 4- or 3-hydroxyproline [1]. There are two sources of proline in the cells. The first one is synthesis from glutamate (Glu) catalyzed by 1-pyrroline-5-carboxylate (P5C) synthase and P5C reductase with P5C as an intermediate product. P5C is formed from both Glu and Pro, due to interconvertibility of this amino acids [2, 3]. Second source of Pro constitute protein degradation products, hydrolyzed by extracellular and intracellular proteases. Among products of protein degradation are imidodipeptides containing C-terminal proline or hydroxyproline [4]. Bond between any amino acid and Pro is resistant to hydrolytic action of most proteases. Only prolidase—cytosolic peptidase is able to hydrolyze imidodipeptides and release Pro [5]. In vivo prolidase is present in many different cell types, including erythrocytes [6], contributing to maintain physiologic level of Pro in blood and extracellular matrix. However, the impact of extracellular Pro on collagen biosynthesis is not fully understood. The reason is that collagen biosynthesis assay is based on radioactive Pro incorporation into collagenous proteins and the tracer is diluted by Pro. Therefore, in this study, we employed Western blot for type I collagen, the most abundant collagen in tissues.

Pro released from imidodipeptides can be reused for protein synthesis, e.g., collagen or metabolized to P5C through proline oxidase, known also as proline dehydrogenase (POX/PRODH) [7, 8]. Prolidase was found to be a rate limiting factor of collagen biosynthesis at least in certain conditions like fibrotic process [9], experimental aging of fibroblasts [10], fibroblasts chemotaxis [11], and cell surface integrin receptor ligation [12]. Interestingly, prolidase was also found as a ligand of epidermal growth factor receptor (EGFR), inducing the receptor signaling [13]. Prolidase activity is also implicated in regulation of some transcription factors. Nuclear factor-κB (NF-κB) transcriptional activity is negatively correlated with prolidase activity [14]. In opposite, hypoxia inducible factor-1 (HIF-1) transcriptional activity positively correlates with prolidase activity. The mechanism of this process is due to Pro-dependent inhibition of HIF-1α degradation [15]. It shows that Pro as a product of prolidase-catalyzed reaction may contribute to the transcriptional action of these factors. In fact, Pro is reported to increase HIF-1α expression and transcriptional activity of HIF-1 in cancer cells [15]. Both NF-κB and HIF-1α regulate collagen biosynthesis on transcriptional and posttranslational levels, respectively [14–18]. The specific purpose of this study was to evaluate the impact of extracellular Pro on collagen, NF-κB, HIF-1α, POX, EGFR, IGF-IR expressions as well as prolidase activity and some signaling proteins in human skin fibroblasts cultured in medium with or without Gln.

## Materials and methods

L-glycyl-proline, L-proline, horseradish peroxidase conjugated anti-mouse IgG (A4416), anti-rabbit IgG (A9169) and anti-goat IgG (A5420) antibodies, bacterial collagenase, 3-(4,5-di-methylthiazole-2-yl)-2,5-diphenyltetrazolium bromide (MTT), monoclonal (mouse) anti-pERK1/2 (M8159), polyclonal (rabbit) anti-IκB-α (I0505) and anti-β-actin (A2066) antibodies, tetrahydro-2-furoic acid (FA), L-4-thiazolidinecarboxylic acid (TA) were provided by Sigma Corp., USA., as were most other chemicals and buffers used. Dulbecco’s minimal essential medium (DMEM), penicillin, streptomycin, and fetal bovine serum (FBS) used in cell culture were products of Gibco, USA. Nitrocellulose membrane (0.2 μm), sodium dodecylsulphate (SDS), polyacrylamide, molecular weight standards, and Coomassie Briliant Blue R-250 were received from Bio-Rad Laboratories, USA. L-5[^3^H] proline (28 Ci/mmol) was purchased from Amersham, UK. Polyclonal (goat) anti-collagen type I, chain α_1_ (COL1A1) (sc-8784) and anti-PRODH/POX antibodies (sc-376401), monoclonal (mouse) anti-p53 (sc-126) antibody, polyclonal (rabbit) anti-IGF-IR (sc-712), anti-EGFR (sc-03), anti-NFκB p65 (sc-372), and anti-α_2_-integrin (sc-9089) antibodies were the products of Santa Cruz Biotechnology Inc., USA. Polyclonal (rabbit) anti-ERK1/2 (9102), monoclonal (rabbit) anti-pAkt (Thr308) (2965), anti-Akt (4685) antibodies were the products of Cell Signaling Inc., USA. Monoclonal (mouse) anti-β_1_ integrin (610468), anti-HIF-1α (610959) antibodies were obtained from Becton, Dickinson Co., USA. Polyclonal (rabbit) anti-prolidase (ab86507) antibody was the product of Abcam, UK. Alexa Fluor 594 anti-mouse (A11032) and anti-rabbit (A11037) polyclonal secondary antibody, and Hoechst 33342 were received from Invitrogen, Carlsbad, CA.

### Tissue culture

All studies were performed on normal human skin fibroblasts CCD25Sk (ATCC^®^ CRL1474™), that were purchased from American Type Culture Collection, Manassas, VA, USA. Reference cells, endometrial adenocarcinoma cells (Ishikawa cell line), and breast adenocarcinoma cells (MCF-7) were received from Sigma Corp., USA). The cells were maintained in DMEM supplemented with 10% fetal bovine serum (FBS), 50 U/ml penicillin, 50 μg/ml streptomycin at 37 °C in a 5% CO_2_ incubator. Cells were counted in hemocytometer and cultured at 1 × 10^5^ cells per well in 2 ml of growth medium in 6-well plates (Costar). Cells reached confluence at day 6 and in most cases such cells were used for assays. Cells were used in the 8th to 14th passages.

### Cell viability assay

The assay was performed according to the method of Carmichael [19] using 3-(4,5-di-methylthiazole-2-yl)-2,5-diphenyltetrazolium bromide (MTT). The cells were cultured for 24 and 48 h with various concentrations of Pro in six-well plates, washed three times with PBS, and then incubated for 4 h in 1 ml of MTT solution (0.5 mg/ml of PBS) at 37 °C. The medium was removed and 1 ml of 0.1 mol/l HCl in absolute isopropanol was added to attached cells. Absorbance of converted dye in living cells was measured at a wavelength of 570 nm. Viability of Pro-treated cells was calculated as a percent of control cells.

### DNA biosynthesis assay

To examine the effect of Pro on fibroblast proliferation, the cells were plated in 24-well tissue culture dishes at 1 × 10^5^ cells/well with 1 ml of growth medium. After 48 h (1.6 ± 0.1 × 10^5^ cells/well), the plates were incubated with various concentrations of Pro and 0.5 μCi of [^3^H] thymidine for 24 h at 37 °C. The radioactivity of incorporated tracer into DNA was measured in a scintillation counter [20].

### Collagen production

Incorporation of radioactive precursor into proteins was measured after labeling of confluent cells (cultured in growth medium with Pro) for the last 24 h with 5[^3^H] proline (5 μCi/ml, 28 Ci/mM) as described previously [21]. Incorporation of tracer into collagen was determined by digesting proteins with purified *Clostridium histolyticum* collagenase, according to the method of Peterkofsky et al. [22]. Results are shown as combined values for cell plus medium fractions.

### SDS-PAGE

Slab SDS/PAGE was used, according to the method of Laemmli [23], using 10% SDS–polyacrylamide gel.

### Western immunoblot analysis

After SDS-PAGE, the gels were allowed to equilibrate for 5 min in 25 mmol/l Tris, 0.2 mol/l glycine in 20% (v/v) methanol. The protein was transferred to 0.2 μm pore-sized nitrocellulose at 100 mA for 1 h by using a BIO-RAD Trans-Blot SD Semi-Dry Electrophoretic Transfer Cell. The nitrocellulose was incubated with primary antibodies at dilution 1:1000 in 5% dried milk in TBS-T (20 mmol/l Tris-HCl buffer, pH 7.4, containing 150 mmol/l NaCl and 0.05% Tween 20) overnight at 4 °C. In order secondary peroxidase conjugated antibodies were added at concentration 1:5000 in TBS-T and incubated for 30 min slowly shaking. Then nitrocellulose was washed with TBS-T (5 × 5 min). Bound antibodies were detected by enhanced chemiluminescence using Amersham ECL Western blotting detection reagents. The intensity of the bands was quantified by densitometric analysis using apparatus for gel documentation BioSpectrum Imaging System (UVP, USA) and presented in arbitral units normalized for β-actin.

### Determination of prolidase activity

The activity of prolidase was determined according to the method of Myara et al. [24]. Protein concentration was measured by the method of Lowry et al. [25]. Enzyme activity was reported as nanomoles of proline released from synthetic substrate, during one minute per milligram of supernatant protein of cell homogenate.

### Immunofluorescence

Fibroblasts were plated in BD Falcon 96-well black/clear bottom tissue culture plates optimized for imaging applications at 1 × 10^5^ cells per well. After 24 h incubation, the cells were rinsed with PBS and fixed with a 4% formaldehyde solution at room temperature for 15 min. After fixation, the cells were washed three times with PBS and permeabilized with a 0.1% Triton X-100 solution at room temperature for 5 min. Then, the cells were washed twice with PBS, and non-specific binding was blocked (5% nonfat dry milk, 10% heat-inactivated goat serum in 0.025% Tween 20/PBS, 1 h incubation at room temperature). After that time, the cells were rinsed, incubated with anti-HIF-1α mouse monoclonal antibody (Becton, Dickinson Co., USA; 1:100) for 1 h at room temperature. Antibody was diluted in antibody dilution buffer (1% nonfat dry milk in 0.025% Tween 20/PBS). Then the cells were washed three times with PBS and incubated with a fluorescent (Alexa Fluor 594) anti-mouse secondary antibody (Invitrogen, Carlsbad, CA) for 60 min in the dark. After washing, the nuclei were stained with Hoechst 33342 (2 μg/ml) and cells were analzed using confocal microscope BD Pathway 855 using a 40 × (0.90 NA) objective.

### Statistical analysis

The results were submitted to statistical analysis using one-way ANOVA followed by Tukey test, accepting *P < 0.05 as significant versus control.

## Results

Treatment of fibroblasts with Pro at concentrations 5 or 10 mM did not influence the viability of the cells. Slight, not significant decrease in the DNA biosynthesis between treated and untreated cells was found (data not shown).

The effect of Pro on collagen biosynthesis in confluent human skin fibroblasts was determined at different time of incubation. Since glutamine (Gln) is an important substrate required for proline biosynthesis [2, 26, 27], we designed conditions of impaired Pro biosynthesis in fibroblasts using Gln-deprived medium. The cells were incubated with complete growth medium containing 10% serum to achieve confluency. Then the cells were pretreated with complete or Gln-deprived medium for 24 h and medium was changed with fresh, containing 5 or 10 mM Pro and 5-[^3^H]-proline (5 μCi/ml, 28 Ci/mM). Incorporation of the radioactivity into proteins susceptible to the action of purified bacterial collagenase was determined according to the method of Peterkofsky et al. [22]. However, we found that the incorporation of radioactive precursor (5[^3^H] proline) into proteins was very low in cells growing in both media (with Gln or without Gln) in presence of Pro suggesting competition between labeled and unlabeled proline in the process of proline incorporation into collagen (data not shown).

**Fig. 1:**
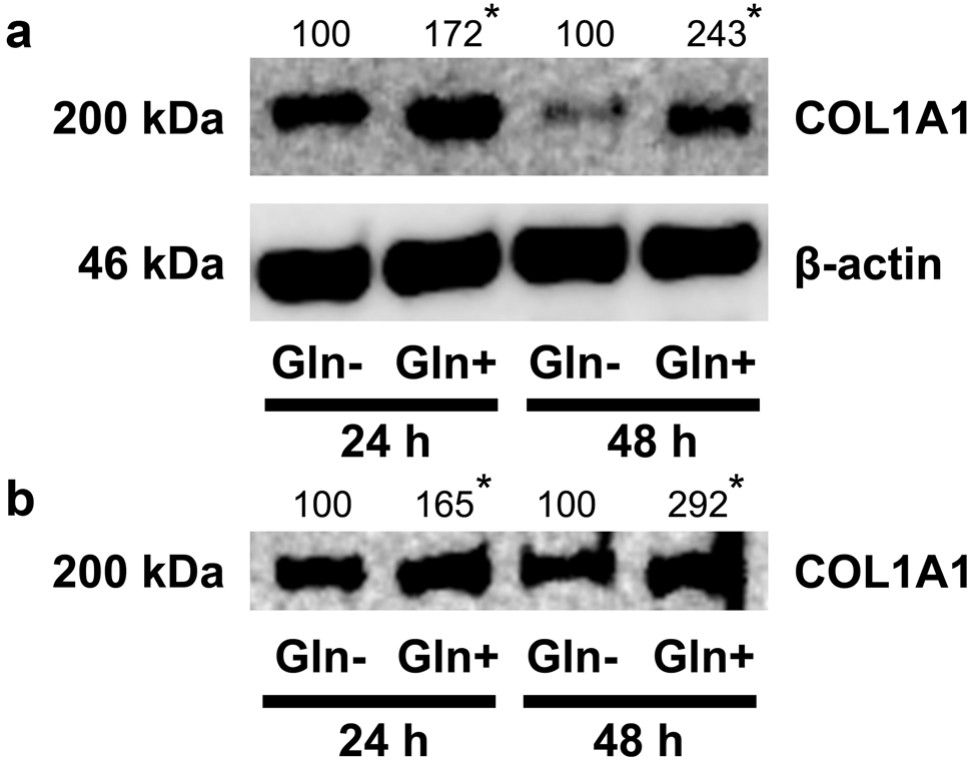
Western blot analysis for collagen type I chain α_1_ (COL1A1) in fibroblasts (**a**) and media (**b**) after incubation of cells for 24 and 48 h in medium with and without Gln. Media were collected and cells were harvested and counted in hemocytometer. The mean values of six pooled cell homogenate extracts from three separate experiments done in duplicate are presented, normalized to β-actin (for intracellular collagen) and collagen/cell number ratio for extracellular collagen, and expressed as percentage of control cells. The intensity of the bands was quantified by densitometric analysis. Densitometry was done with BioSpectrum Imaging System and presented as arbitrary units. *Numbers* indicate results of densitometric analysis of proteins with normalization to *β*-actin levels. Significant (*P* < 0.05) changes in band intensities compared with control are indicated by *asterisk*. The same amount of supernatant protein (20 μg) was run in each lane

Therefore, western blot analysis of α_1_ subunit of collagen type I (COL1A1) in the cell homogenates and culture media, after 24 and 48 h of fibroblasts treatment with or without Gln was performed. As can be seen in Fig. 1a, expression of COL1A1 in fibroblasts and cultured medium (Fig. 1b) is much higher in cells growing in Gln containing medium, compared to cells cultured in medium without Gln. Collagen expression in cells treated with Gln for 24 and 48 h reached 172 and 243% of control value (cells cultured without Gln), respectively. Similarly, expression of collagen in culture medium of these cells achieved 165 and 292% of control value, respectively.

**Fig. 2:**
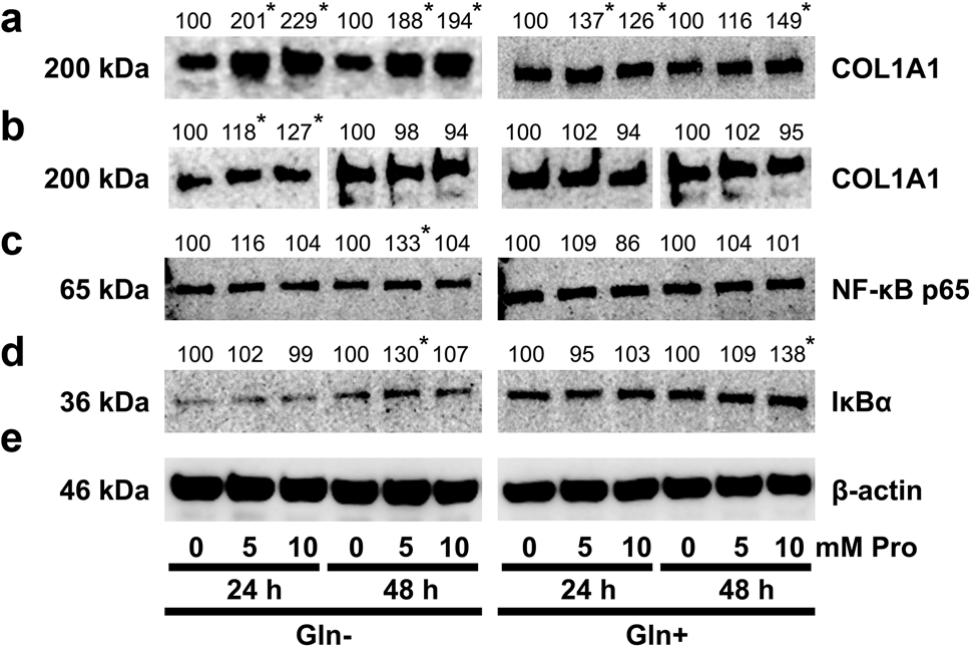
Western blot analysis for COL1A1 in fibroblasts (**a**) and media (**b**), incubated for 24 and 48 h with different concentrations of Pro in media, with and without Gln, and western blot analysis for NF-κB p65 (**c**) and IκBα (**d**) in fibroblasts incubated in the same conditions. The mean values of six pooled cell homogenate extracts from three separate experiments done in duplicate are presented. The intensity of the bands was quantified by densitometric analysis. Densitometry was done with BioSpectrum Imaging System and presented as arbitrary units. *Numbers* indicate results of densitometric analysis of proteins with normalization to *β*-actin levels. Significant (*P* < 0.05) changes in band intensities compared with control are indicated by *asterisk*. The same amount of supernatant protein (20 μg) was run in each lane. The expression of β-actin served as a control for protein loading (**e**)

Treatment of the cells with 5 or 10 mM Pro in both Gln-supplemented and Gln-deprived medium for 24 and 48 h induced expression of COL1A1 (Fig. 2a). However, removal of Gln from medium augmented Pro-dependent increase in expression of collagen type I in fibroblasts. Fibroblasts growing in medium without Gln containing 5 or 10 mM Pro produced much more (about twice) COL1A1, during the course of experiment compared to control. Increase in the expression of COL1A1 in the cells was accompanied by slight increase in secretion of collagen to the medium, but only after 24 h of incubation of the cells (Fig. 2b).

Pro-dependent stimulation of collagen production was not related to the expression of NF-κB p65 (Fig. 2c), the known inhibitor of collagen gene expression [28] in fibroblasts, growing in Gln containing medium. Moreover, in all conditions Pro was unable to decrease IκB-α expression (Fig. 2d), suggesting that canonical NF-κB activation is not induced by proline [29, 30].

**Fig. 3:**
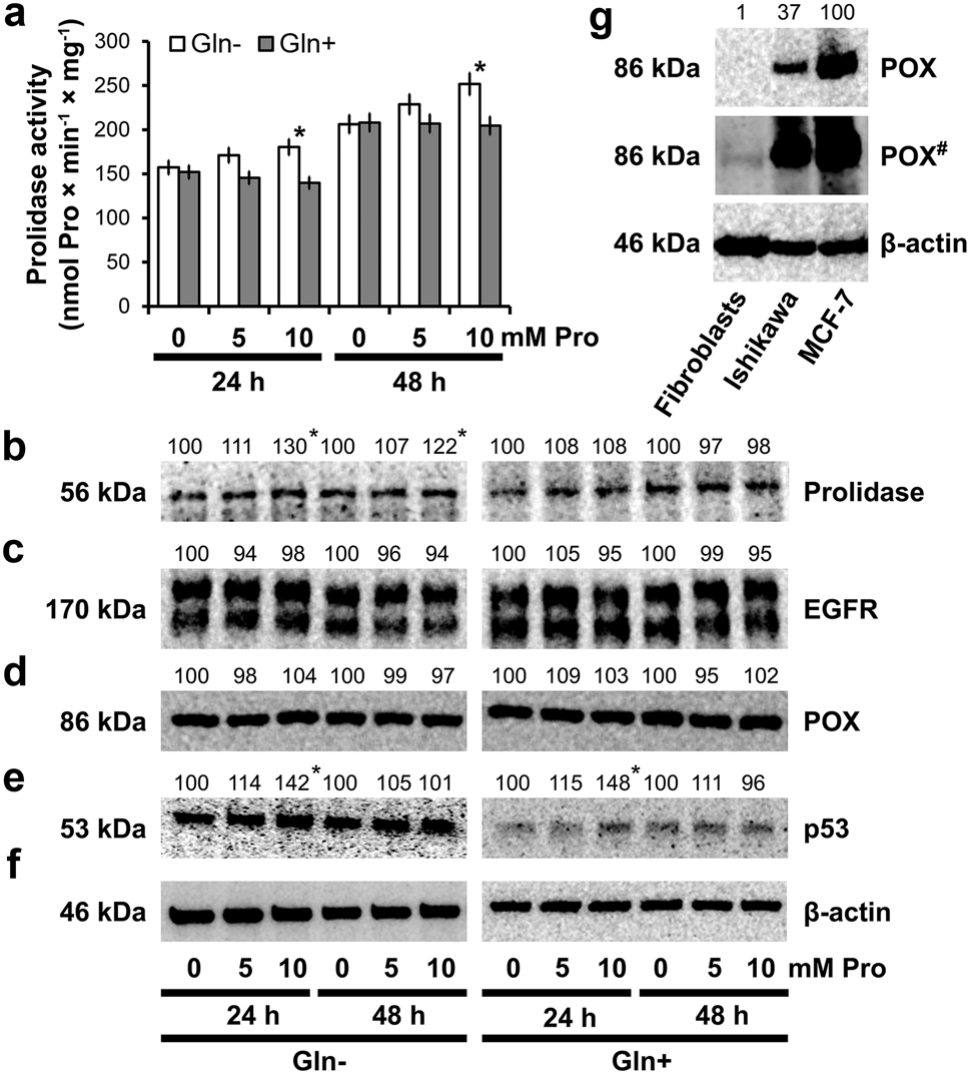
Prolidase activity (**a**) in confluent human skin fibroblasts incubated for 24 and 48 h in the medium with and without Gln, containing 10% FBS and different concentrations of Pro. The results present the mean values from six assays ± S.D. *P < 0.05. Western blot analysis for prolidase (**b**), EGFR (**c**), POX (**d**), and p53 (**e**) in fibroblasts incubated for 24 and 48 h with different concentrations of Pro. Western blot analysis for POX in fibroblasts and cancer cell lines Ishikawa and MCF-7 (**g**), # longer exposure. The mean values of six pooled cell homogenate extracts from three separate experiments done in duplicate are presented. The intensity of the bands was quantified by densitometric analysis. Densitometry was done with BioSpectrum Imaging System and presented as arbitrary units. *Numbers* indicate results of densitometric analysis of proteins with normalization to *β*-actin levels. Significant (*P* < 0.05) changes in band intensities compared with control are indicated by *asterisk*. The same amount of supernatant protein (20 μg) was run in each lane. The expression of β-actin served as a control for protein loading (**f**)

Since prolidase is involved in collagen biosynthesis by recycling Pro from protein degradation, the enzyme activity was evaluated. We have found that Pro increased the enzyme activity by about 15–20%, during the course of experiment and the process was more pronounced in Gln-deprived medium (Fig. 3a). This increase in the enzyme activity in cells cultured in Gln-deprived medium was correlated with the enzyme expression (Fig. 3b).

Since prolidase is involved in regulation of epidermal growth factor receptor (EGFR) signaling [13], the expression of this receptor was measured by Western blot analysis. However, Pro had no significant effect on the expression of EGFR (Fig. 3c).

Proline is utilized not only in protein synthesis but also in process of Pro oxidation to P5C, catalyzed by mitochondrial proline oxidase (POX). The enzyme expression was determined in fibroblasts, MCF-7 and endometrial adenocarcinoma cells, as a reference cell lines. We found very low expression of POX in fibroblasts, using Western blot with enhanced chemiluminescence as a method of detection (Fig. 3g).

Pro did not change expression of POX in fibroblasts (Fig. 3d); however, it increased p53 expression (Fig. 3e) known inducer of POX gene transcription [31].

**Fig. 4:**
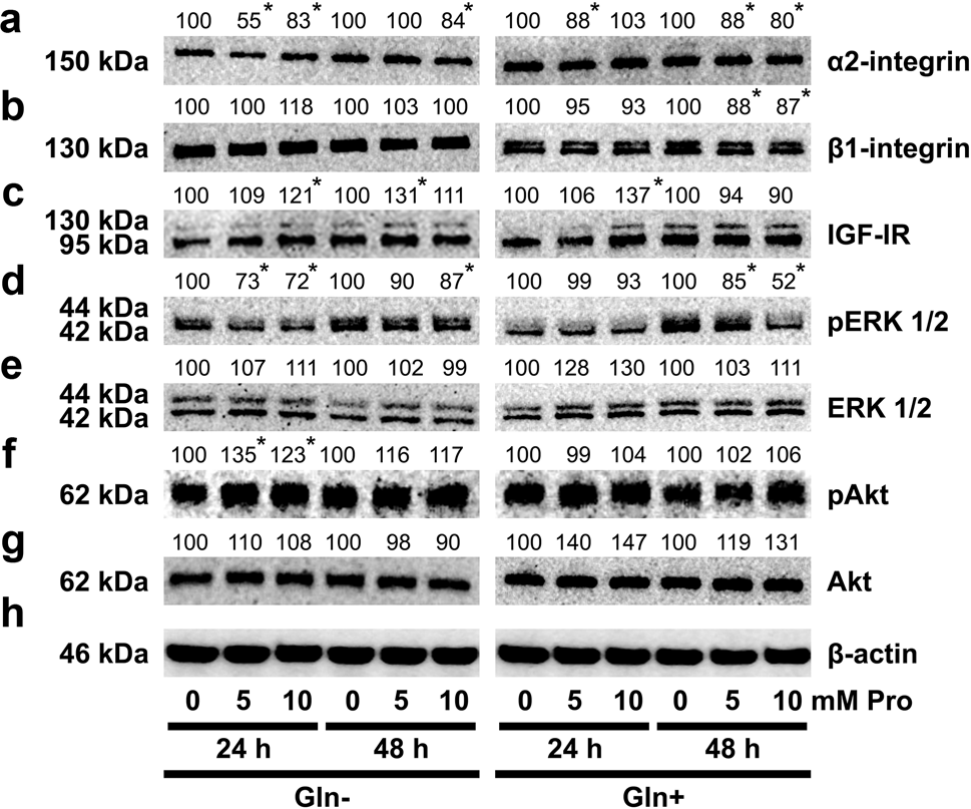
Western blot analysis for α_2_ integrin (**a**), β_1_-integrin subunit (**b**), IGF-I receptor (**c**), pERK1/2 (**d**), ERK1/2 (**e**), pAkt (**f**), and Akt (**g**) in fibroblasts incubated for 24 and 48 h in medium with and without Gln, in the presence of different concentrations of Pro. The mean values of six pooled cell homogenate extracts from three separate experiments done in duplicate are presented. The intensity of the bands was quantified by densitometric analysis. Densitometry was done with BioSpectrum Imaging System and presented as arbitrary units. *Numbers* indicate results of densitometric analysis of proteins with normalization to *β*-actin levels. Significant (*P* < 0.05) changes in band intensities compared with control are indicated by *asterisk*. The same amount of supernatant protein (20 μg) was run in each lane. The expression of β-actin served as a control for protein loading (**h**)

Collagen biosynthesis and prolidase activity were previously shown to be regulated due to the signal induced by activated α_2_β_1_ integrin receptor [12, 32] as well as IGF-IR [33]. Therefore, the expression of α_2_β_1_ integrin receptor (receptor for type I collagen) and IGF-IR were measured by Western immunoblot analysis. As can be seen in Fig. 4a, b, treatment of fibroblasts with 5 or 10 mM Pro contributed to decrease in the expression of α_2_ and β_1_ integrin subunits, particularly in cells growing in medium with Gln. As shown on Fig. 4c, IGF-I receptor expression was increased in Pro-treated cells growing in medium with Gln, after 24 h incubation. This stimulating effect was not observed after 48 h. In fibroblasts, growing in Gln-deprived medium treatment of fibroblasts with 5 or 10 mM Pro contributed to increase in the expression of IGF-I receptor to about 109 and 121% after 24 h of incubation and 131 and 111% after 48 h of incubation, compared to the control cells (Fig. 4c).

We found that in fibroblasts growing in medium with Gln, 5 or 10 mM Pro decreased expression of phosphorylated ERK1/2 to about 93% after 24 h and to 85 and 52% after 48 h, compared to the control cells (Fig. 4d). In growth medium without Gln, 24 and 48 h treatment of fibroblasts with 5 or 10 mM Pro contributed to decrease in the expression of phosphorylated ERK1/2 to about 70% after 24 h and 87% after 48 h, compared to the control cells (Fig. 4d).

In Pro-treated cells growing in medium without Gln, an expression of pAkt was increased (Fig. 4f), suggesting that in the experimental conditions, Akt protein represent signaling molecule that respond to Pro action.

**Fig. 5:**
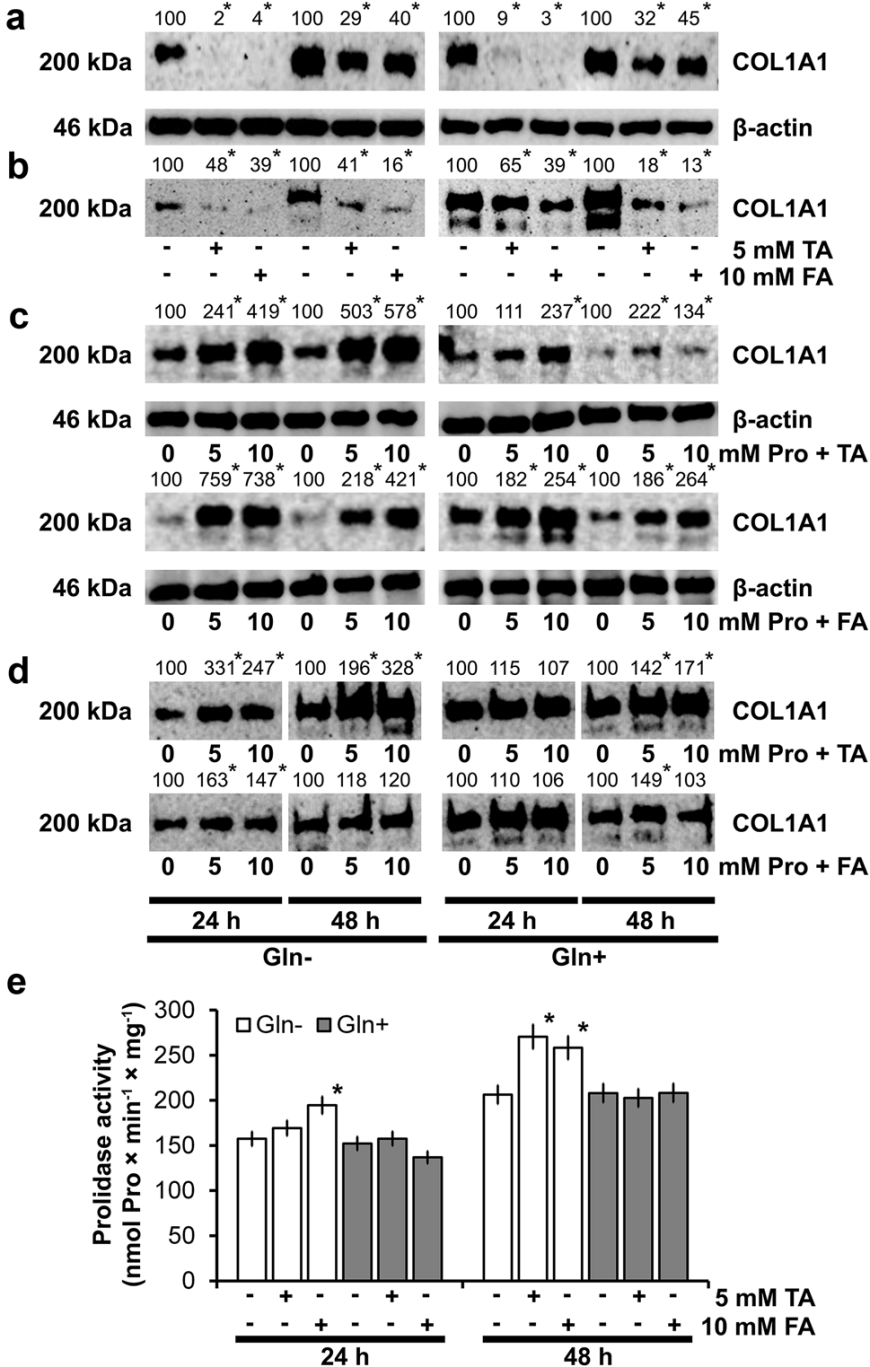
Western blot analysis for COL1A1 in fibroblasts (**a**) and media (**b**), incubated for 24 and 48 h with different concentrations of Pro in media, with and without Gln. Western blot analysis for COL1A1 in fibroblasts (**c**) and COL1A1 secreted to medium (**d**). Cells were incubated in the presence of different concentrations of Pro with 5 mM TA or 10 mM FA. The mean values of six pooled cell homogenate extracts from three separate experiments done in duplicate are presented. The intensity of the bands was quantified by densitometric analysis. Densitometry was done with BioSpectrum Imaging System and presented as arbitrary units. *Numbers* indicate results of densitometric analysis of proteins with normalization to *β*-actin levels. Significant (*P* < 0.05) changes in band intensities compared with control are indicated by *asterisk*. The same amount of supernatant protein (20 μg) was run in each lane. The expression of β-actin (**a**, **c**) served as a control for protein loading. Prolidase activity (**e**) in confluent human skin fibroblasts incubated for 24 and 48 h in the medium with and without Gln, containing 10% FBS and 5 mM TA or 10 mM FA. The results present the mean values from six assays ± S.D. *P < 0.05

To study the specificity of proline impact on collagen biosynthesis, we used proline analogues: tetrahydro-2-furoic acid (FA) and thiazolidine-4-carboxylic acid (TA). Due to structural similarity to proline, these compounds can probably substitute proline in collagen biosynthesis. It has been also documented that they impair proline oxidation by POX [34–36]. Based on cytotoxicity of both compounds, we selected concentration 10 mM of FA and 5 mM of TA. As shown in Fig. 5a, after 24 and 48 h incubation of fibroblasts in medium with Gln, expression of COL1A1 was decreased by analogues in similar way: to 30% by TA and to 40% by FA, compared to control. In cells growing in medium without Gln structural analogues of Pro almost completely inhibited expression of COL1A1. Thus, the inhibitory effect of Pro analogues on collagen biosynthesis is stronger in cells with impaired proline biosynthesis. Similar process was observed on expression of COL1A1 secreted to medium (Fig. 5b).

Next, we assessed whether Pro addition can affect inhibitory effect of TA and FA on collagen expression. Pro was added after 2 h pretreatment of cells with proline analogues and cultured for 24 and 48 h. We observed that in both, presence or absence of Gln in media, Pro reversed the inhibitory effect of its analogues. The effect of Pro was strongest in fibroblasts growing in medium without Gln (Fig. 5c). However, Pro-induced collagen expression in media (Fig. 5d) was less pronounced, than in the cells (Fig. 5c).

Regarding that molecules similar to Pro, like Cbz-Pro inhibit prolidase activity [14], we checked influence of FA and TA on prolidase. In cells growing in medium with Gln, both compounds did not affect prolidase activity after 48 h incubation. In cells growing in medium without Gln, after 48 h of incubation TA and FA increased the enzyme activity to 130 and 125% of control values, respectively (Fig. 5e).

**Fig. 6:**
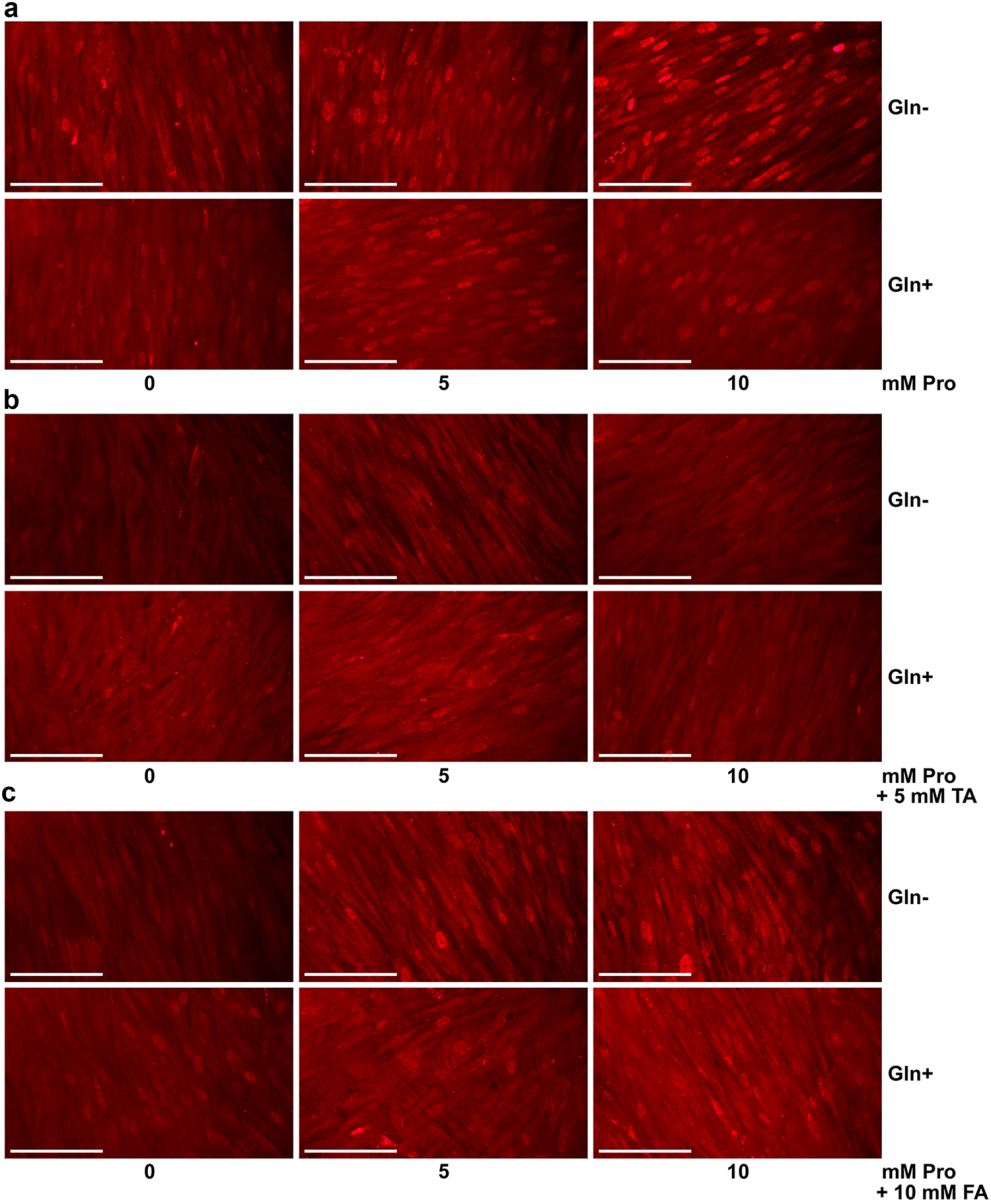
Immunofluorescence staining of HIF-1α in fibroblasts incubated for 48 h with different concentrations of Pro in media, with and without Gln (**a**), with 5 mM TA addition (**b**) or with 10 mM FA addition (**c**). *Scale bar* 100 μm

Pro is reported to increase HIF-1α expression in cancer cells [15]. Heterodimeric factor HIF-1α stimulates the posttranslational hydroxylation of procollagen prolyl residues [37], necessary to obtain the correct spatial structure of collagen [1]. Recent studies indicate inhibition of transcription of type I collagen genes by HIF-1 [18]. Therefore, the expression of HIF-1α in Pro-treated cells was evaluated by bioimaging. It was found increased staining for HIF-1α in Pro-treated cells, cultured in both media for 48 h, especially with 10 mM of Pro in medium without Gln, compared to control cells (Fig. 6, panel a). An addition of TA and FA to Pro-treated cells in both medium abolishes increased staining for HIF-1α, but FA was less effective (Fig. 6, panel b, c).

## Discussion

In this report, we provide evidence that extracellular Pro has significant, but relatively little impact on collagen expression in cells cultured in standard conditions in medium containing Gln. The conclusion is supported by other authors, showing that an addition of proline has failed to increase collagen biosynthesis in fibroblasts and other cells [38–40]. In contrast, extracellular Pro drastically increased collagen expression in the cells cultured in Gln-free medium. The mechanism of this process is due to the availability of substrate (Gln) for intracellular Pro biosynthesis.

Pro is formed from glutamate (Glu) which is produced from Gln. The main source of Gln in the body is Glu in muscle that is converted to Gln by glutamine synthase. The enzyme is widely distributed in tissues and was reported also in fibroblasts [41].

Gln was referred as an inducer of collagen gene transcription [42]. Culture medium (DMEM) contains high concentration of Gln (4 mM) that after conversion may support also intracellular pool of Pro. Therefore, we evaluated the effect of extracellular Gln deprivation on collagen expression in human skin fibroblasts. In fact, Gln deprivation diminished collagen expression, while Pro partly counteracted this effect. The action of proline in this process cannot be linked to conversion of Pro to Gln because activity of proline oxidase (POX) in fibroblasts is very low. Therefore, we suggest that Gln deprivation impaired Pro biosynthesis contributing to relative proline deficiency that was partly normalized by Pro addition. Thus, fibroblasts need exogenous Gln, as an intermediate of proline, required for effective collagen biosynthesis.

Another source of Pro are imidodipeptides hydrolyzed by prolidase. However, the enzyme activity was only slightly but significantly increased by Pro in the cells cultured in Gln-free medium, suggesting the marginal role of prolidase for proline support in a such conditions. Therefore, we considered another function of prolidase. Previously, it has been suggested that increase in prolidase activity (that release proline from imidodipeptides) contribute to increase in NF-κB p65 expression [14]. This process is important for collagen type I biosynthesis, since transcription of genes coding type I collagen subunits is inhibited by NF-κB [16, 27, 28]. However, we found that extracellular Pro did not affect expression of NF-κB. Therefore, we postulate that extracellular Pro may participate in collagen biosynthesis as a substrate.

The evidence that supply of Pro is essential for the biosynthesis of collagen comes from the experiment showing that proline analogues suppress collagen expression. The mechanism of proline analogues competition with Pro in collagen biosynthesis is well known phenomenon [43]. Impaired ability of fibroblasts to synthesize Pro was shown in the cells incubated in medium without Gln. An addition of exogenous Pro reversed this effect presumably as a result of competition mechanism.

Based on previous studies on exogenous glutamine-induced collagen biosynthesis [40, 44] and present data showing that collagen biosynthesis is not suppressed completely in fibroblasts growing in Gln-deprived medium, we hypothesize that the effect can be associated with synthesis of glutamine/glutamate and recycling of proline by prolidase. Pro is converted in mitochondria by POX to P5C and further to glutamate, ornithine, or proline. POX is expressed ubiquitously in the body, but POX activity was found previously to be undetectable in fibroblasts [45]. In the present study we noticed very low expression of POX in fibroblasts, which suggest that the proline in fibroblasts is mainly consumed for protein biosynthesis.

**Fig. 7:**
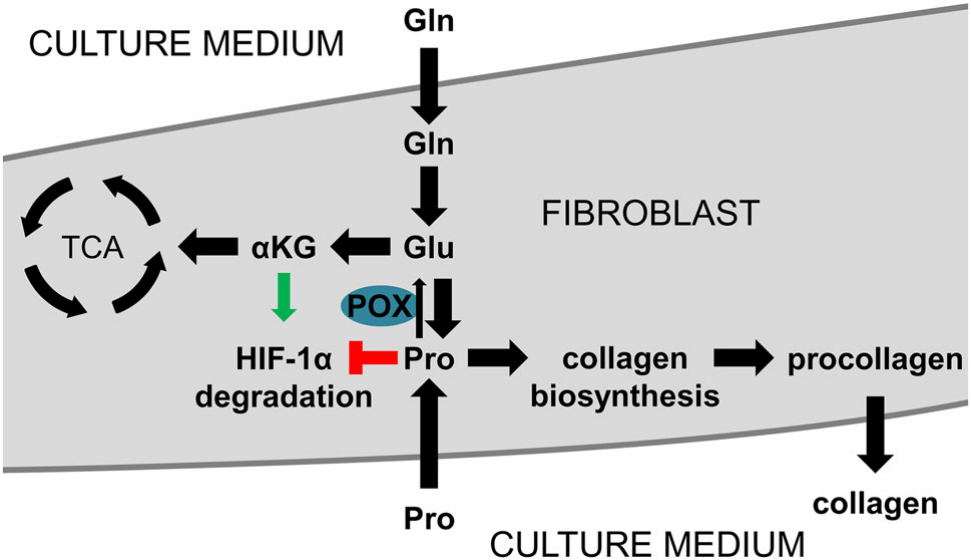
Hypothetical mechanism for the role of exogenous proline in stimulation of type I collagen and HIF-1α expression. Glutamine contained in culture medium is converted in the cells into glutamate and further in mitochondria into α-ketoglutarate, intermediate of tricarboxylic acid cycle. Glutamate is also a source of proline required for collagen biosynthesis. This pathway of proline supply is sufficient to maintain high collagen biosynthesis rate. An addition of exogenous proline results in slight increase in collagen production. Glutamine deprivation from culture medium contributes to decrease in cellular proline content resulting in down-regulation of collagen biosynthesis. In such a condition, exogenous proline restores intracellular proline pool, providing substrate for collagen biosynthesis. The conversion of proline into glutamate is not significant due to low expression of proline oxidase (POX) in fibroblasts. This hypothesis is supported by proline-dependent regulation of HIF-1α transcriptional activity. Translocation of HIF-1α to nucleus is more pronounced in the absence of glutamine since α-ketoglutarate induces HIF-1α hydroxylation and ubiquitin dependent degradation. Therefore, in the absence of glutamine, α-ketoglutarate production is impaired, contributing to upregulation of HIF-1α transcriptional activity. In contrast, in the presence of glutamine, proline-induced HIF-1α transcriptional activity is attenuated. *Pro* proline; *POX* proline oxidase; *Gln* glutamine; *Glu* glutamate; *HIF-1α* hypoxia inducible factor-1α; *αKG* α-ketoglutarate; *TCA* tricarboxylic acid cycle

Pro was referred as a HIF-1α inducing agent in colon cancer cell line RKO [15]. Pro addition prevent hydroxylation of specific proline residue in the oxygen-dependent degradation (ODD) domain of HIF-1α and prevent targeting HIF-1α for ubiquitination and proteasomal degradation via Von Hippel-Lindau (VHL) tumor suppressor protein [46]. RKO cell line expresses POX [47]; thus, it is not clear whether Pro or its metabolites affect HIF-1α expression. We presented that Pro induced HIF-1α translocation to nuclei of fibroblasts (the cells showing low expression of POX). Therefore, stimulation of HIF-1α is not an effect of metabolites of Pro. It is interesting that this process is stronger in the absence of Gln. In fibroblasts Gln is converted to Glu, which is the source of α-KG. It is known that Gln deprivation from the medium results in lowering the concentration of α-KG [48, 49], whereby the cells could become more susceptible to the stabilizing effect of proline on HIF-1α. The hypothesis is outlined in Fig. 7. However, our study on the effect of proline analogues on the expression of HIF-1α provided not expected data. Pro analogues diminished HIF-1α expression in the cells and prevented its upregulation by proline addition. However, Pro-dependent stimulation of intracellular collagen expression in fibroblasts was not affected by proline analogues. Therefore, it seems that this process do not depend on HIF-1. In fact, recent studies documented inhibition of transcription of type I collagen genes by HIF-1 [18]. Nevertheless, the explanation of this effect requires further study.

Proline-dependent increase in HIF-1 expression was preceded by an increase in p53 expression. So far, stimulating effect of proline on p53 expression was not described. It is known that p53 is inducer of POX expression [31, 47]; however, proline did not affect POX expression. Moreover, increased expression of p53 did not affect cell viability suggesting that apoptosis was not induced. The functional significance of proline-dependent regulation of p53 transcriptional activity requires further study.

In view of our data, the signaling pathways induced by IGF-I are also involved in stimulation of intracellular collagen expression by proline. We found that Pro-dependent increase in expression of type I collagen in fibroblasts was accompanied by activation of IGF-IR expression and Akt signaling. In fact, IGF-I is the most potent stimulator of collagen biosynthesis [33, 50] and IGF-I receptor-mediated Akt-dependent signaling pathway induces collagen gene expression [51]. Collagen biosynthesis is regulated also by α_2_β_1_ integrin [12, 32]. However, we found that stimulation of collagen expression in studied conditions is not dependent on α_2_β_1_ integrin signaling.

The results of this study lead to conclusion that availability of glutamine, as a substrate for proline biosynthesis determines the utilization of exogenous proline for collagen biosynthesis.
